# Supplementary material for: The First Cold Atmospheric Plasma Phase I Clinical Trial for the Treatment of Advanced Solid Tumors: A Novel Treatment Arm for Cancer
Source: Cancers (Basel). 2023 Jul 20;15(14):3688. doi: 10.3390/cancers15143688 (PMC10378184; doi:10.3390/cancers15143688)
Supplement: Supplementary file 1 [file cancers-15-03688-s001.zip › Supplementary Figures_Final.pdf]

Supplementary Appendix

This appendix has been provided by the authors to give readers additional information about the work

Supplementary Appendix

Table of Contents to the Supplementary Appendix

I. Supplementary Figures
   a. Figure S1. CHCP Treatment Temperature Measurements
   b. Figure S2. Patient vital signs during surgery
   c. Figure S3. Patient vital signs during surgery
   d. Figure S4. Histopathology of Patient Tissue Samples
   e. Figure S5. Primary Cell Culture of Patient Tumor Samples
   f. Figure S6. TUNEL Assay Analysis
   g. Figure S7. Western blot analysis for apoptotic markers after CHCP treatment on patient tumor samples.
   h. Figure S8. Tumor Cell Death at the surgical margin
II. Supplementary Tables
   a. Table S1. Gene profiling analysis

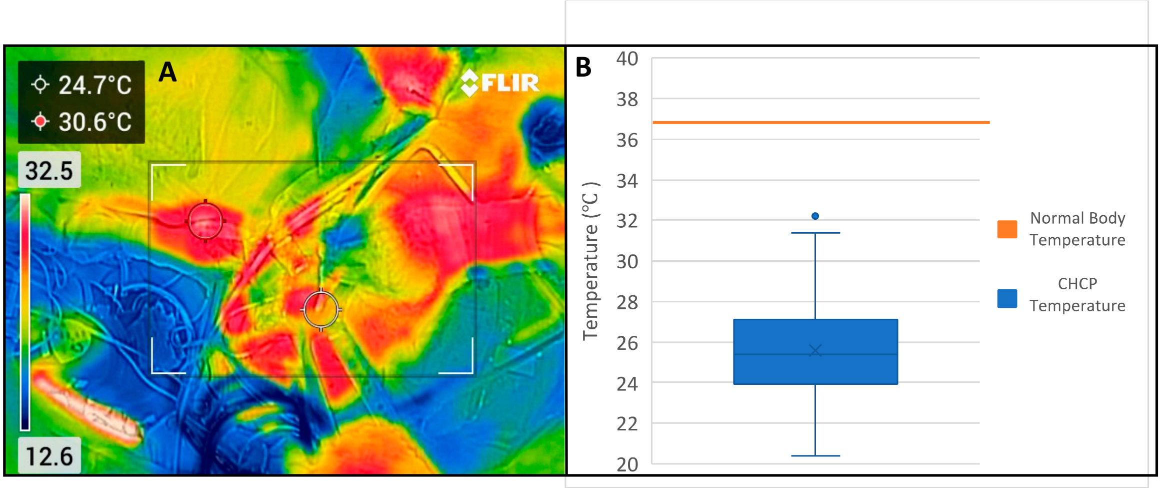

Figure S1. CHCP treatment temperature measurements. (A) A representative Forward Looking InfraRed (FLIR) thermal camera image showing the surgical margin treatment area and surrounding tissue. The CHCP beam temperature is 24.7 °C. (B) Plot of the mean, median, interquartile range of measured CHCP beam temperatures relative to normal body temperature.

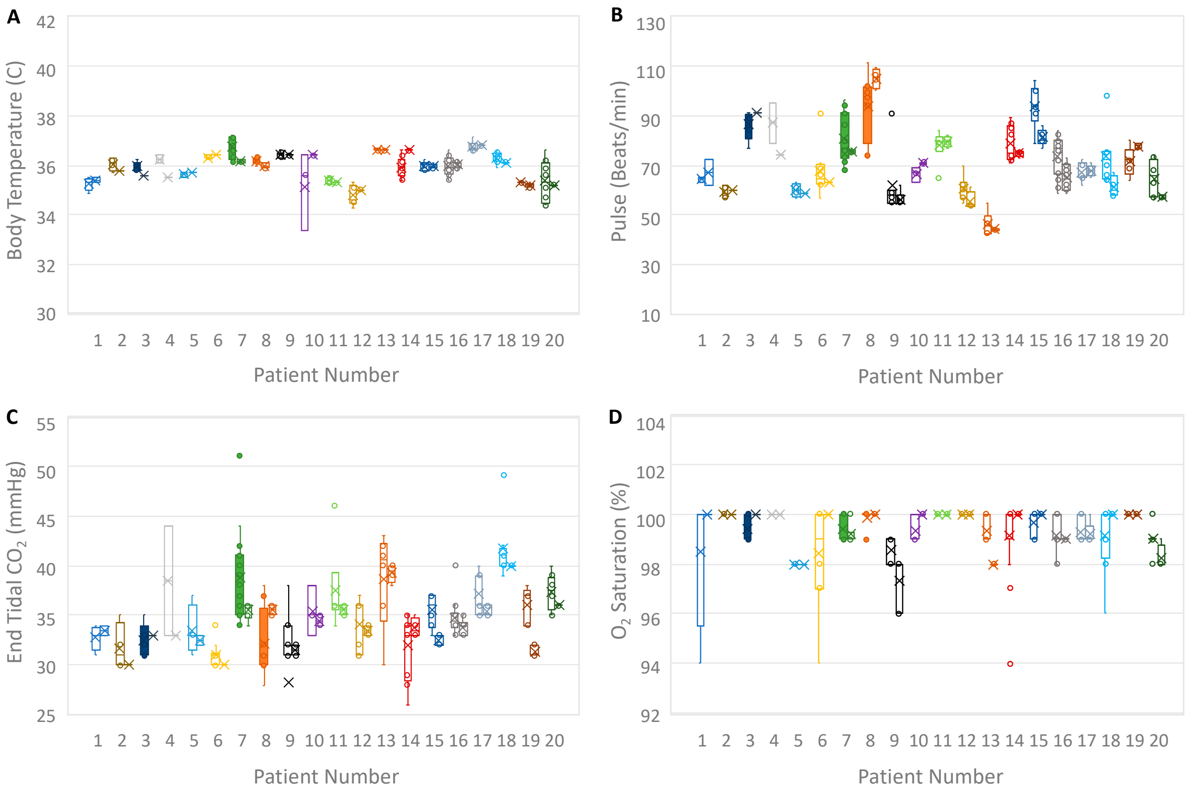

E

**Median blood pressure (IQR) mmHg**	**During CHCP treatment**	**Not during CHCP Treatment**
**Systolic**	105 (94-109)	112 (102-120)
**Diastolic**	62 (55-66)	67 (63-72)

Figure S2. Patient vital signs range in box plots. Throughout the surgery, intraoperative vital signs including (A) body temperature, (B) pulse, (C) End Tidal CO_2 and (D) O_2 Saturation during non-CHCP treatment time (left) and CHCP treatment time (right) for each patient were tracked. No significant changes ($p > 0.05$) in patient vitals were found between CHCP treatment times and non CHCP treatment times. (E) Table showing the average systolic and diastolic blood pressure measured throughout surgery and CHCP treatment. There was no significant difference ($p < 0.05$) between median blood pressure during CHCP compared to not during CHCP.

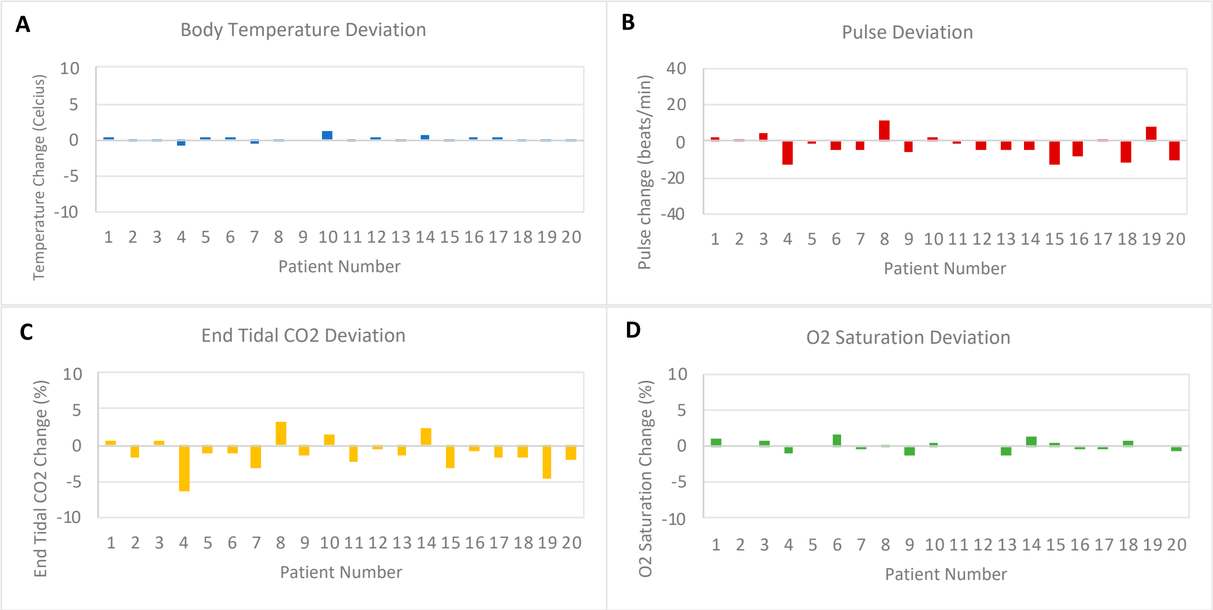

E

Vitals	Δ = CHCP – Non CHCP (Average of 20)
Body Temperature (°C)	-0.003
Pulse (beats/min)	-2.64
End Tidal CO2 (mmHg)	-1.11
O2 Saturation (%)	0.15

Figure S3. Patient vital signs deviation. Intraoperative vital signs including (A) body temperature, (B) pulse, (C) End Tidal CO_2 and (D) O_2 Saturation during CHCP treatment (averaged) compared to non-CHCP treatment time (averaged) for each patient. (E) Table showing the change of intraoperative vitals during CHCP treatment times compared to non CHCP treatment times.

Figure S4. Histopathology of Patient Tissue Samples. Hematoxylin and eosin (H&E) stained slides showing (A) Tumor, (B) Zone Zero, (C) Zone 1, and (D) Normal tissue samples with or without CHCP treatment. Black arrows and yellow arrows point to untreated tumor cells and treated dead tumor cells respectively. Microscopic tumor cells were identified at the surgical margin site (Zone 0) in 5 patients (42%) (R0004, R0005,R0009 R0011 and R0012). Tumor and zone zero tissues show no visible non-thermal damage. Light Micrographs displaying the morphological Spectrum at 63X.

Patient # R0002

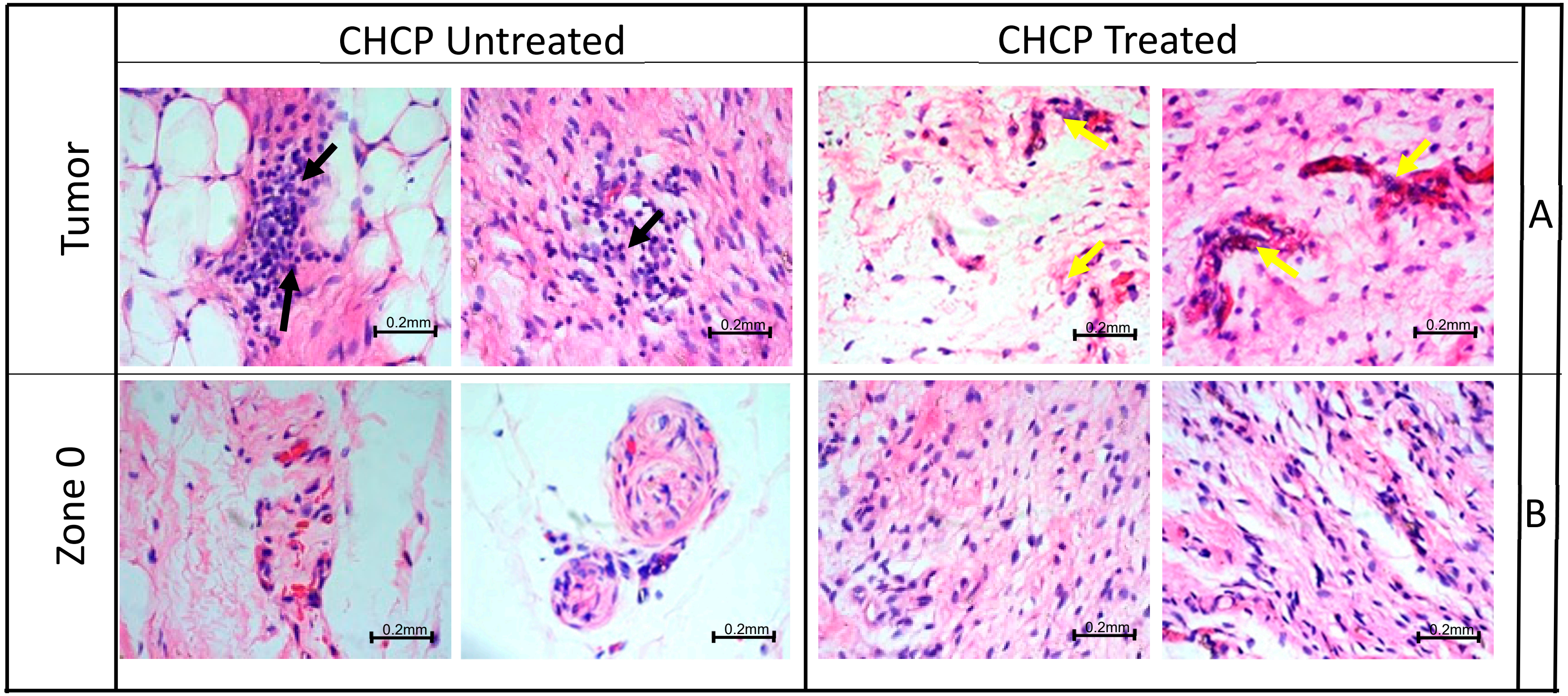

Figure S4. Histopathology of Patient Tissue Samples

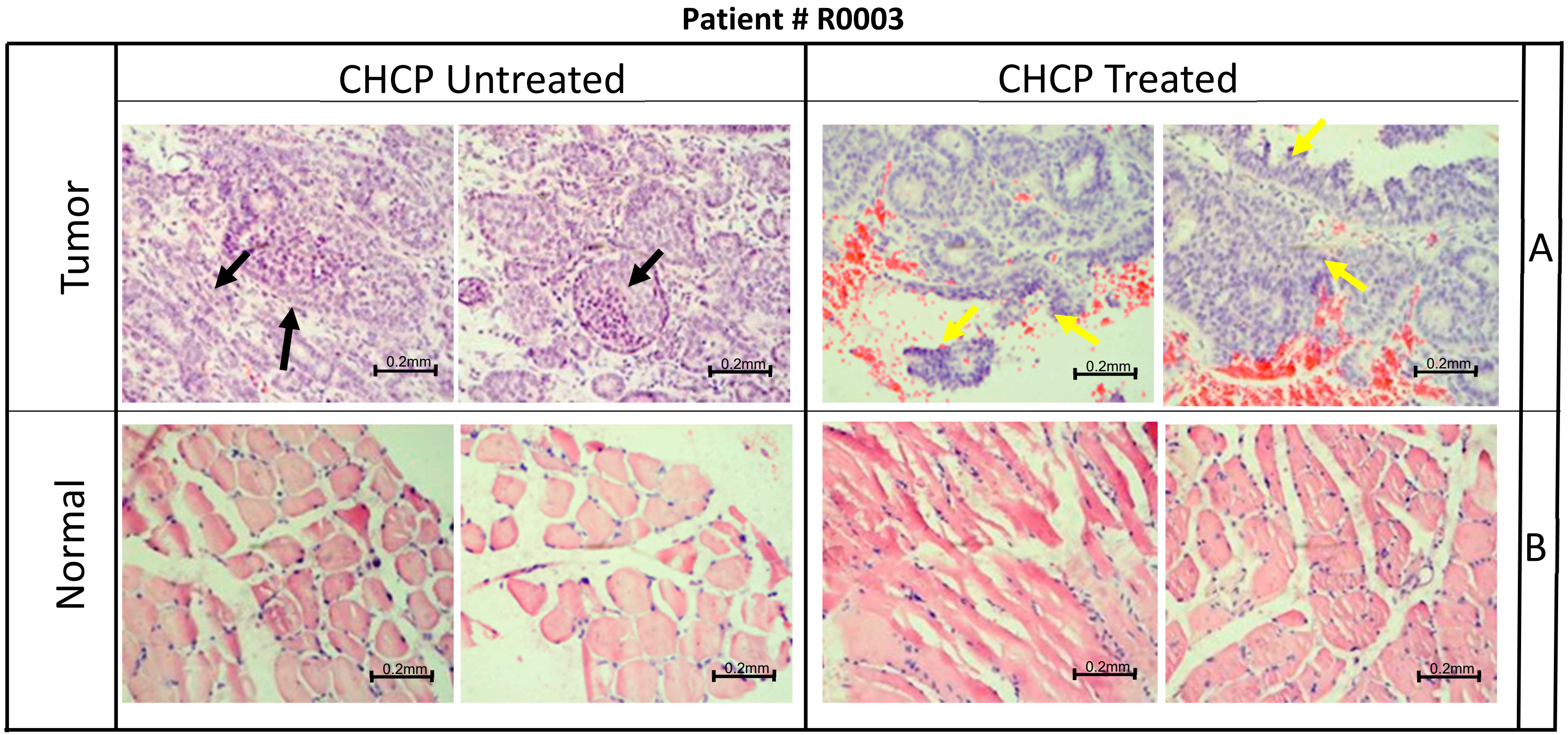

Figure S4. Histopathology of Patient Tissue Samples

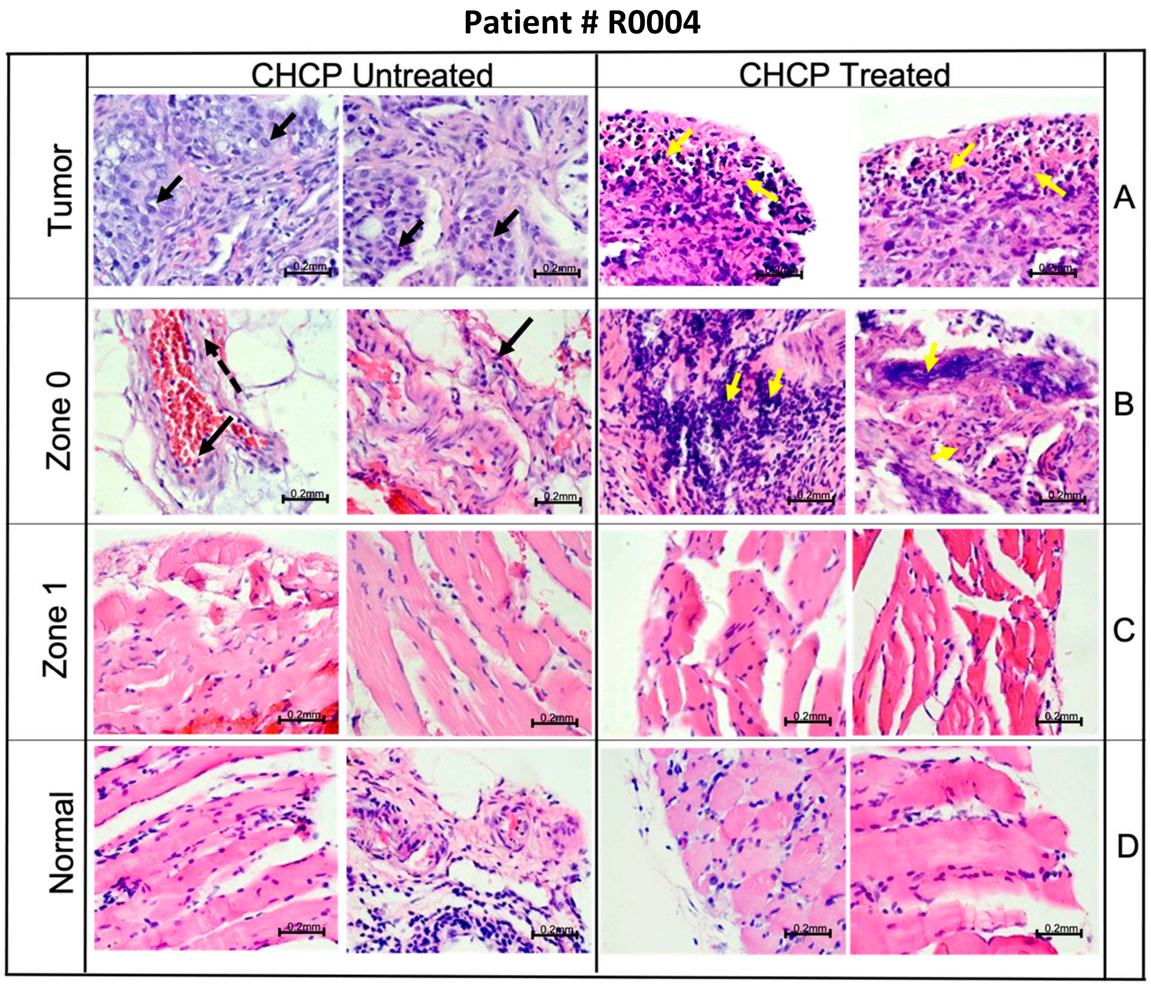

Figure S4. Histopathology of Patient Tissue Samples

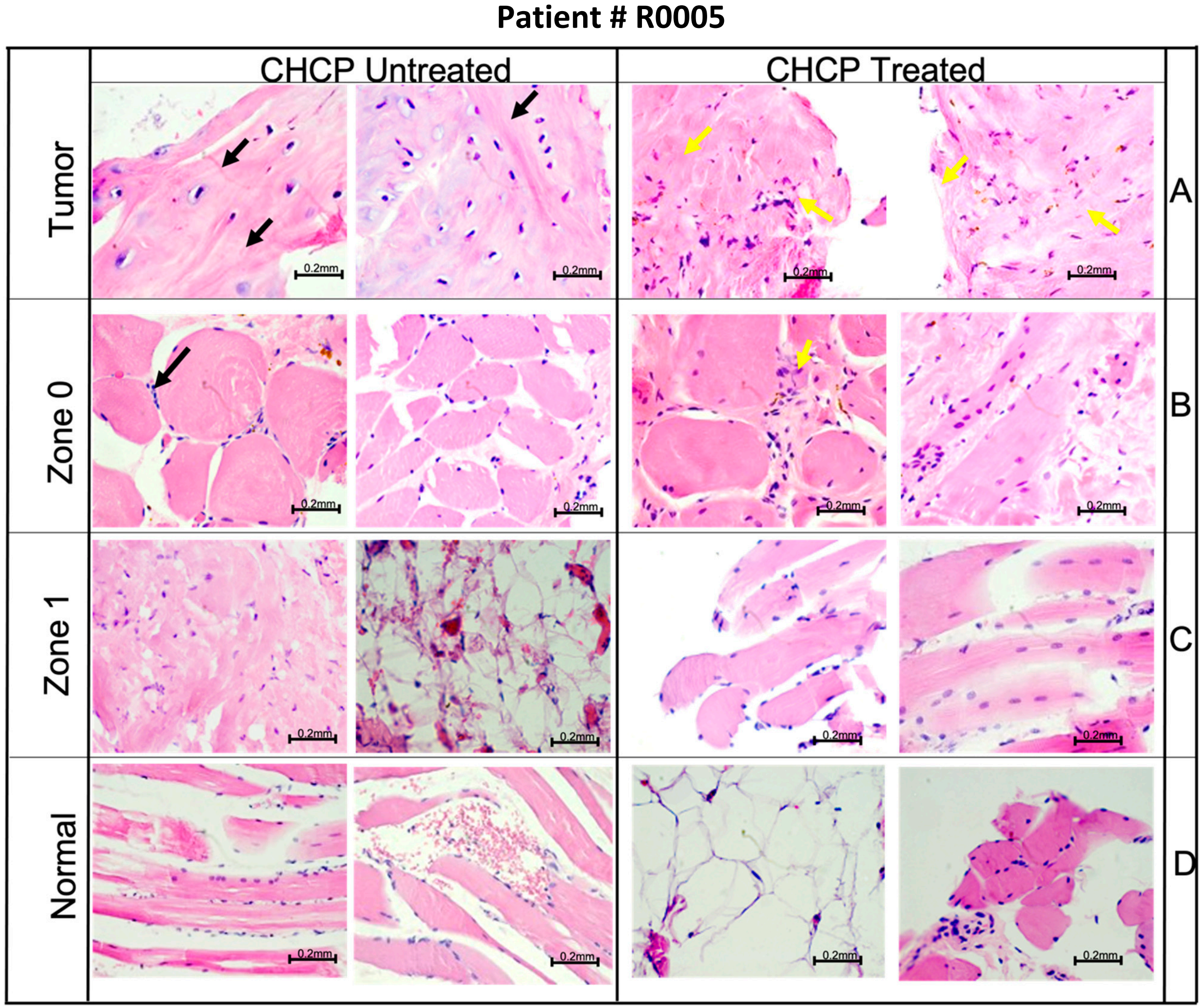

Figure S4. Histopathology of Patient Tissue Samples

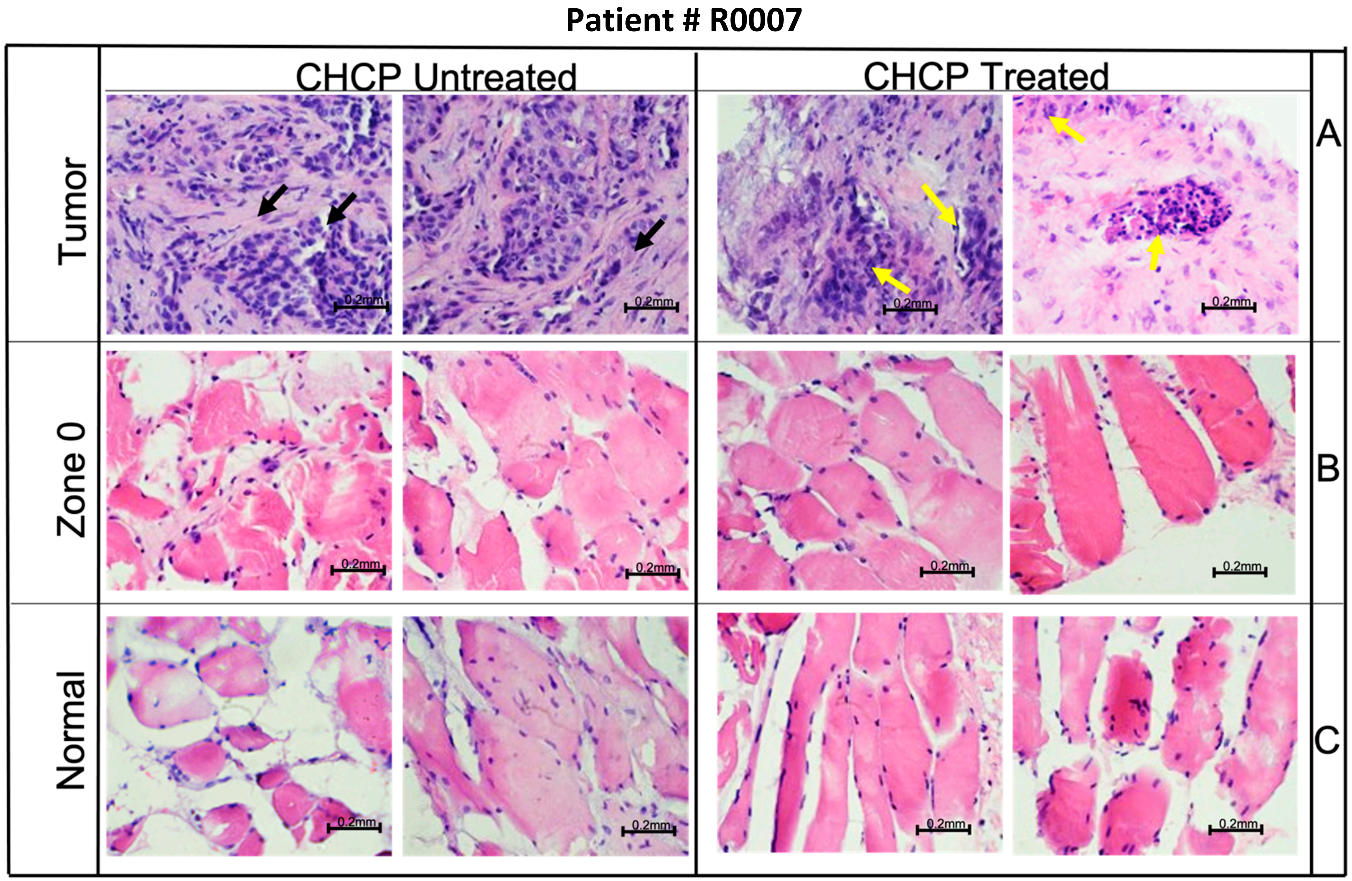

Figure S4. Histopathology of Patient Tissue Samples

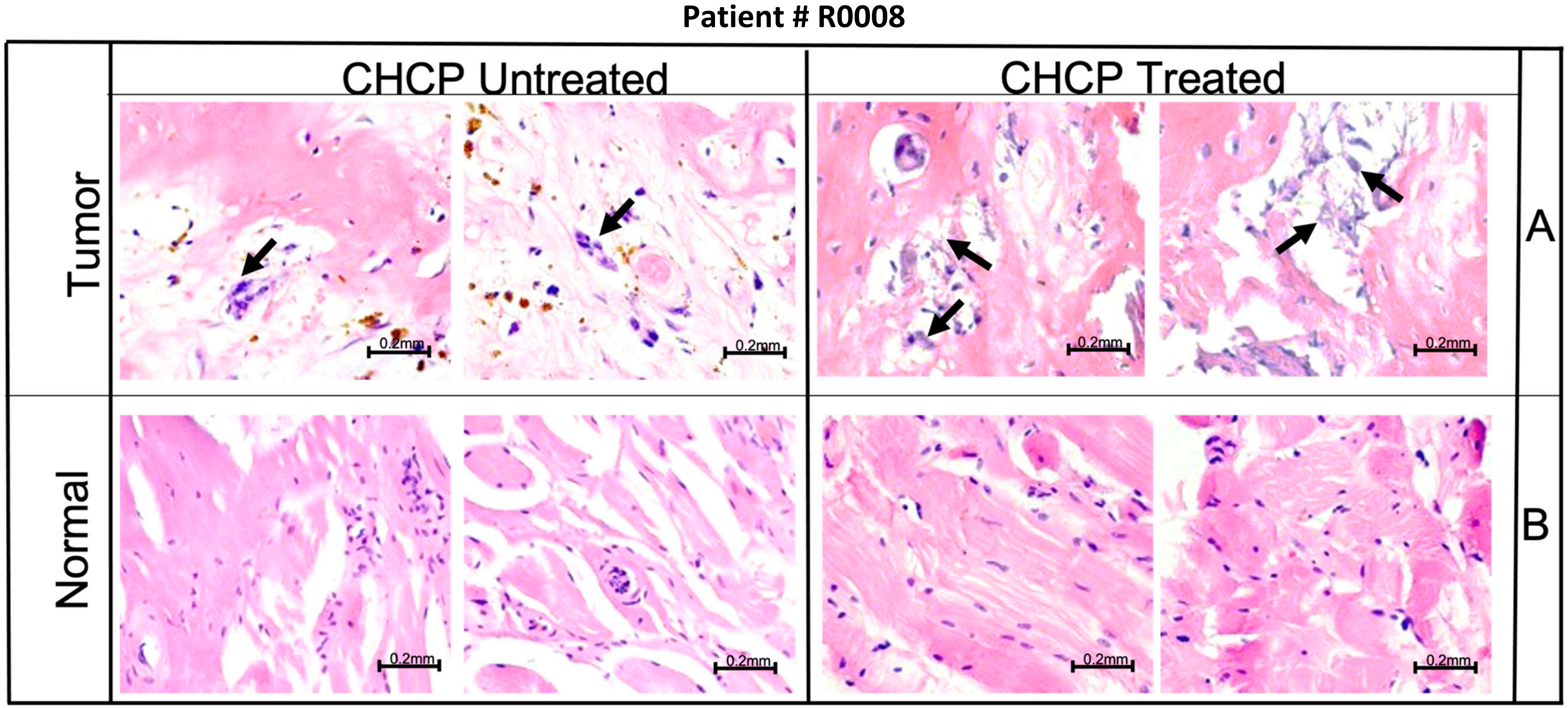

Figure S4. Histopathology of Patient Tissue Samples

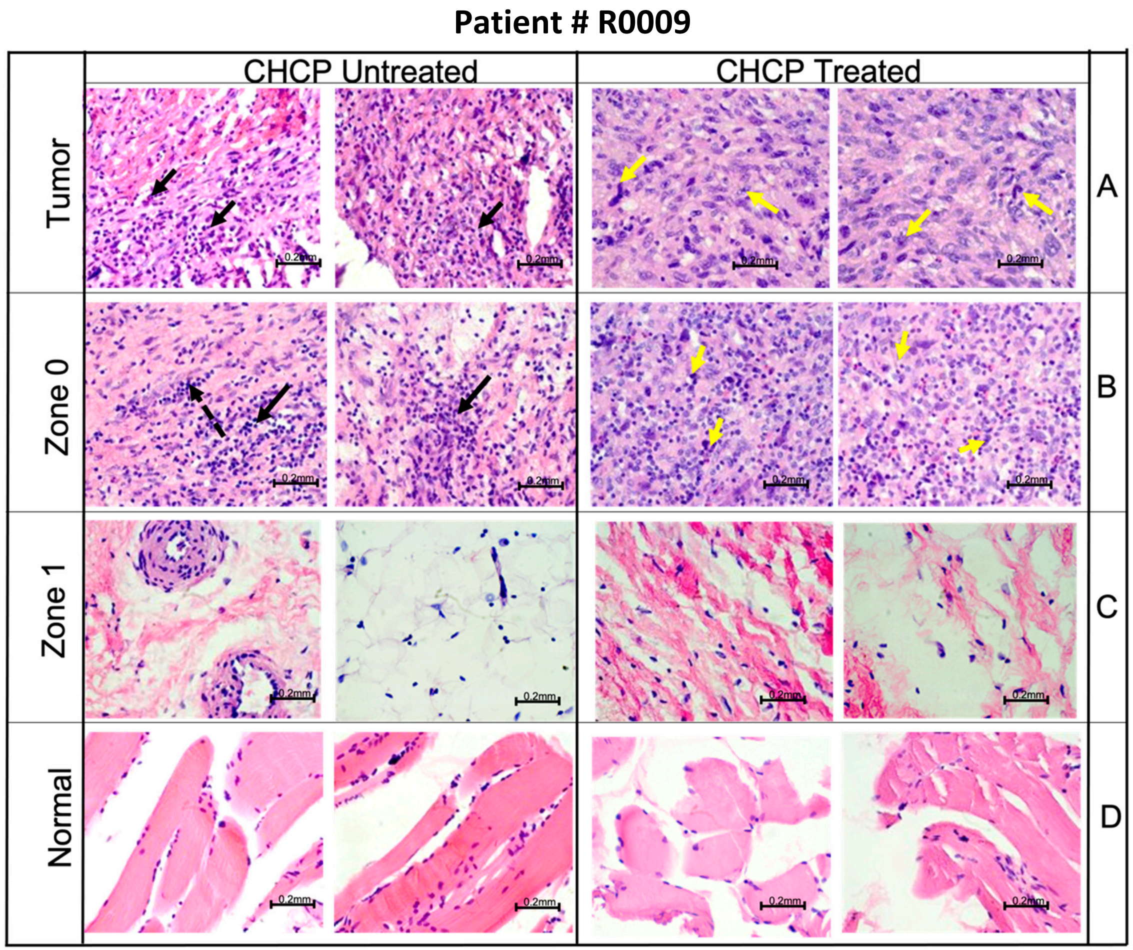

Figure S4. Histopathology of Patient Tissue Samples

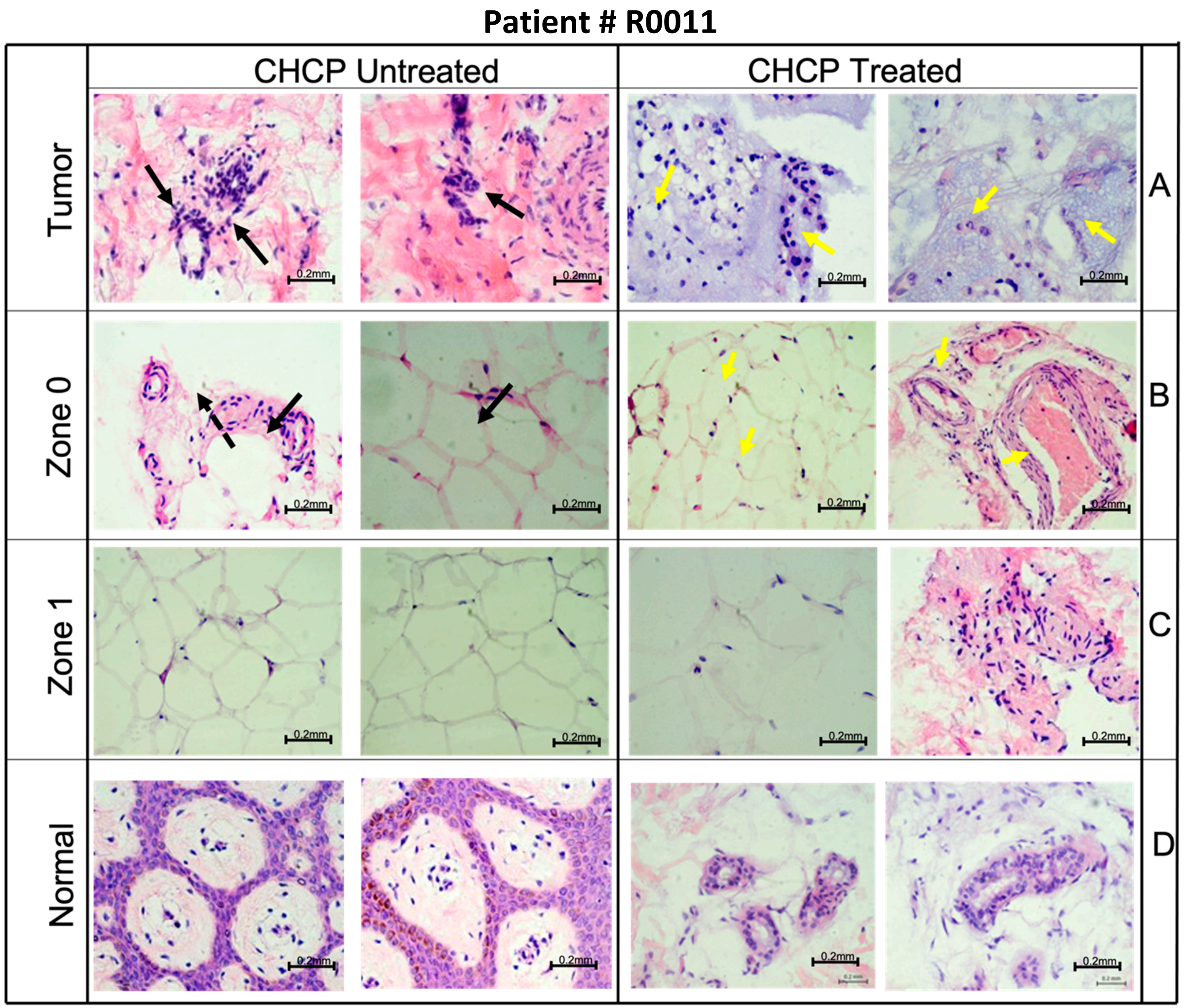

Figure S4. Histopathology of Patient Tissue Samples

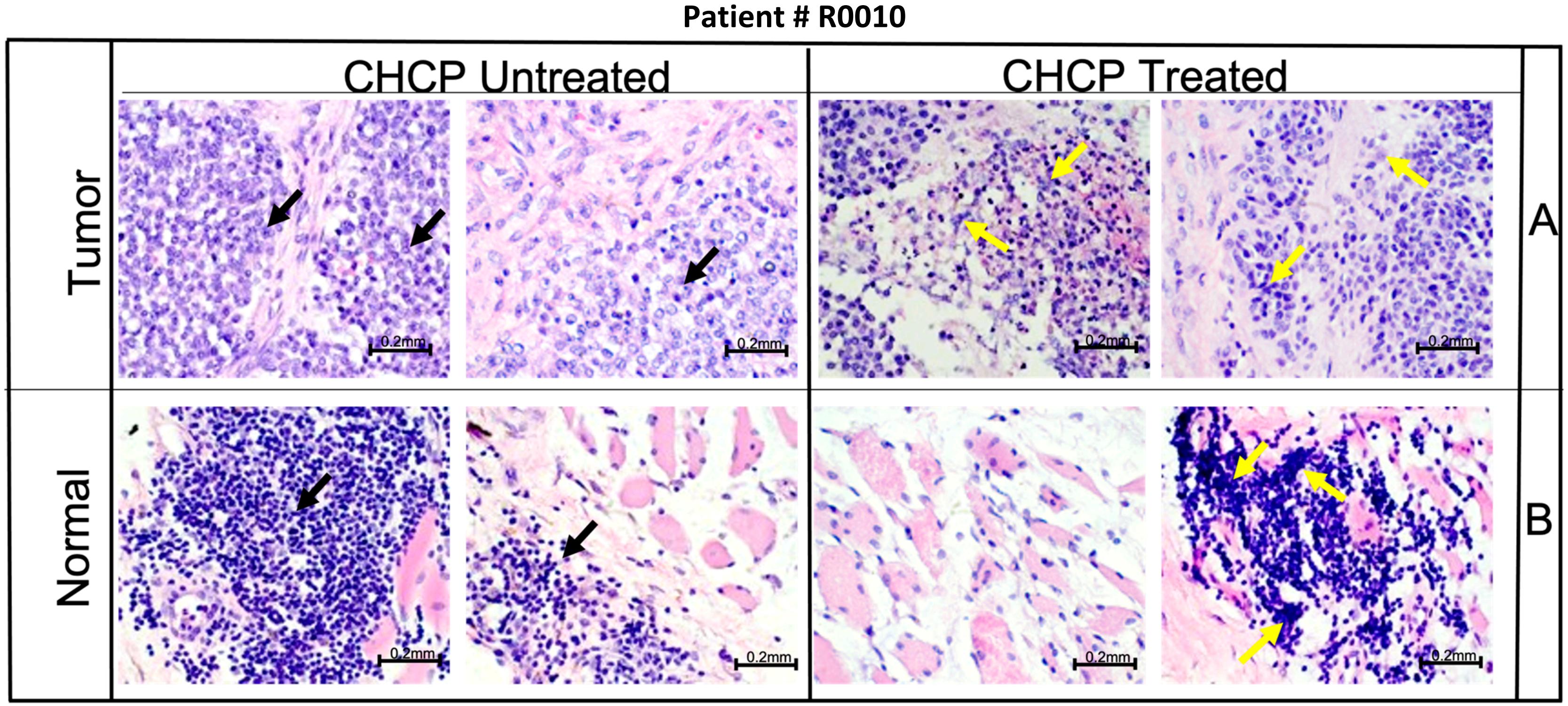

Figure S4. Histopathology of Patient Tissue Samples.

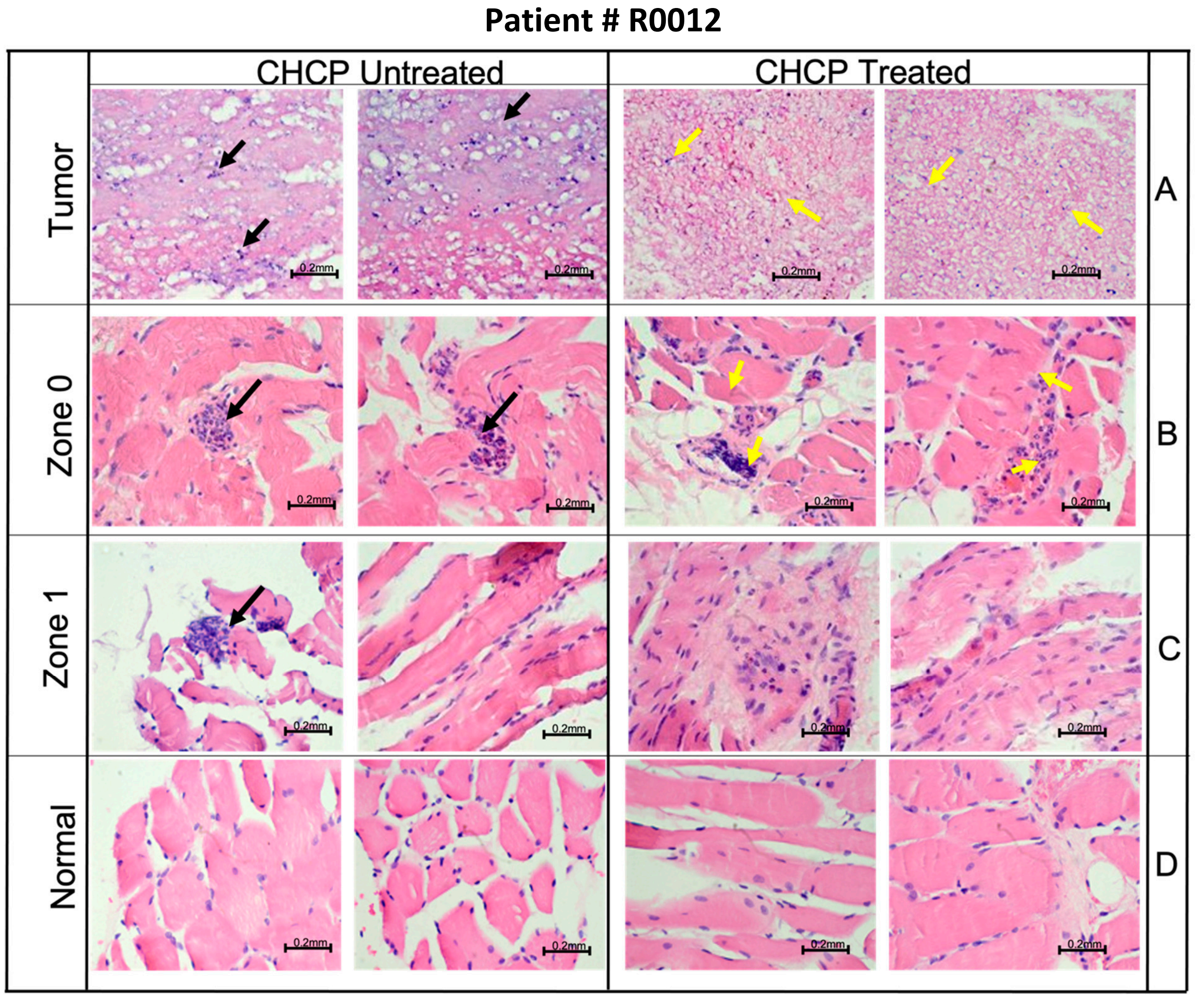

Figure S4. Histopathology of Patient Tissue Samples

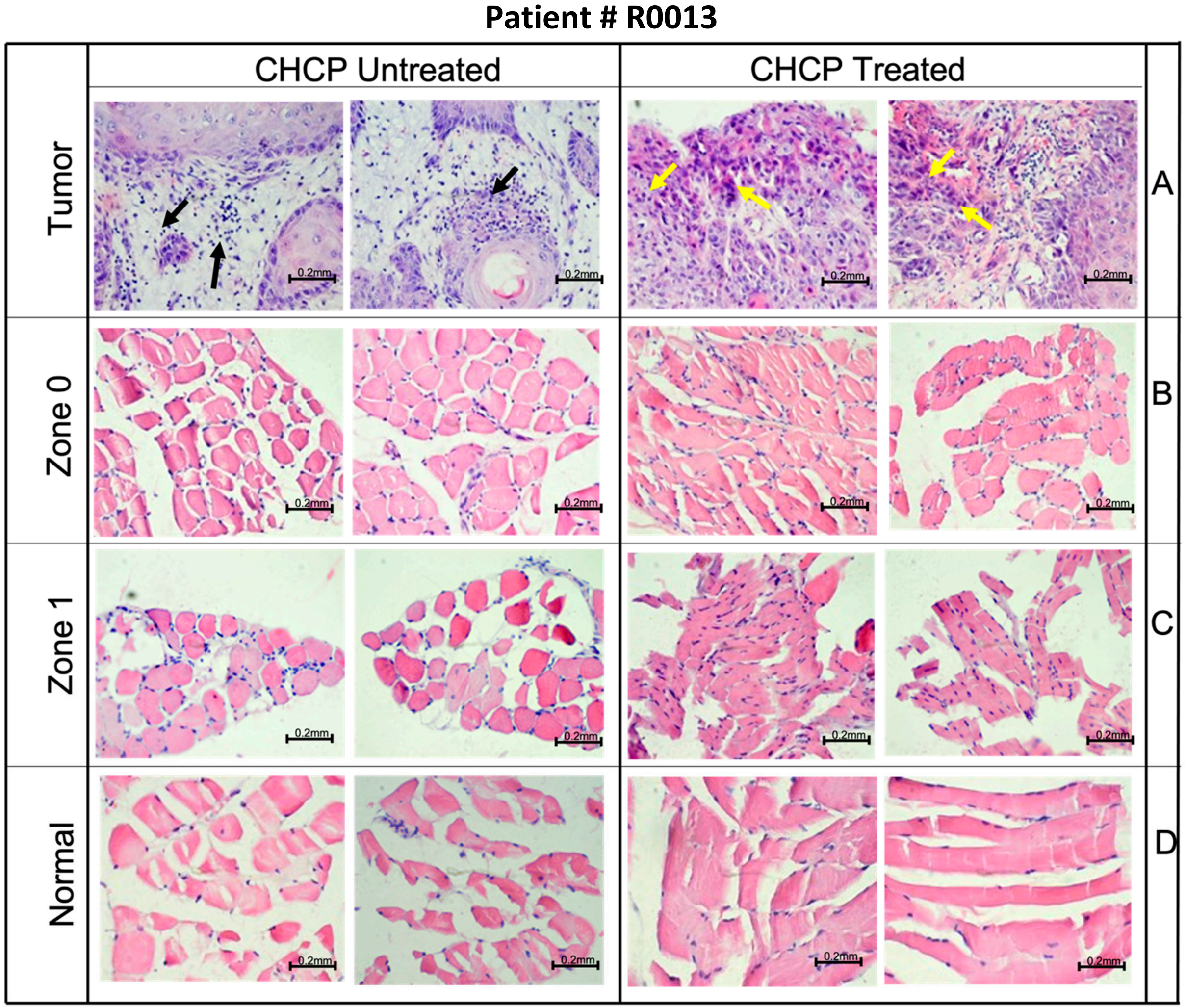

Figure S4. Histopathology of Patient Tissue Samples

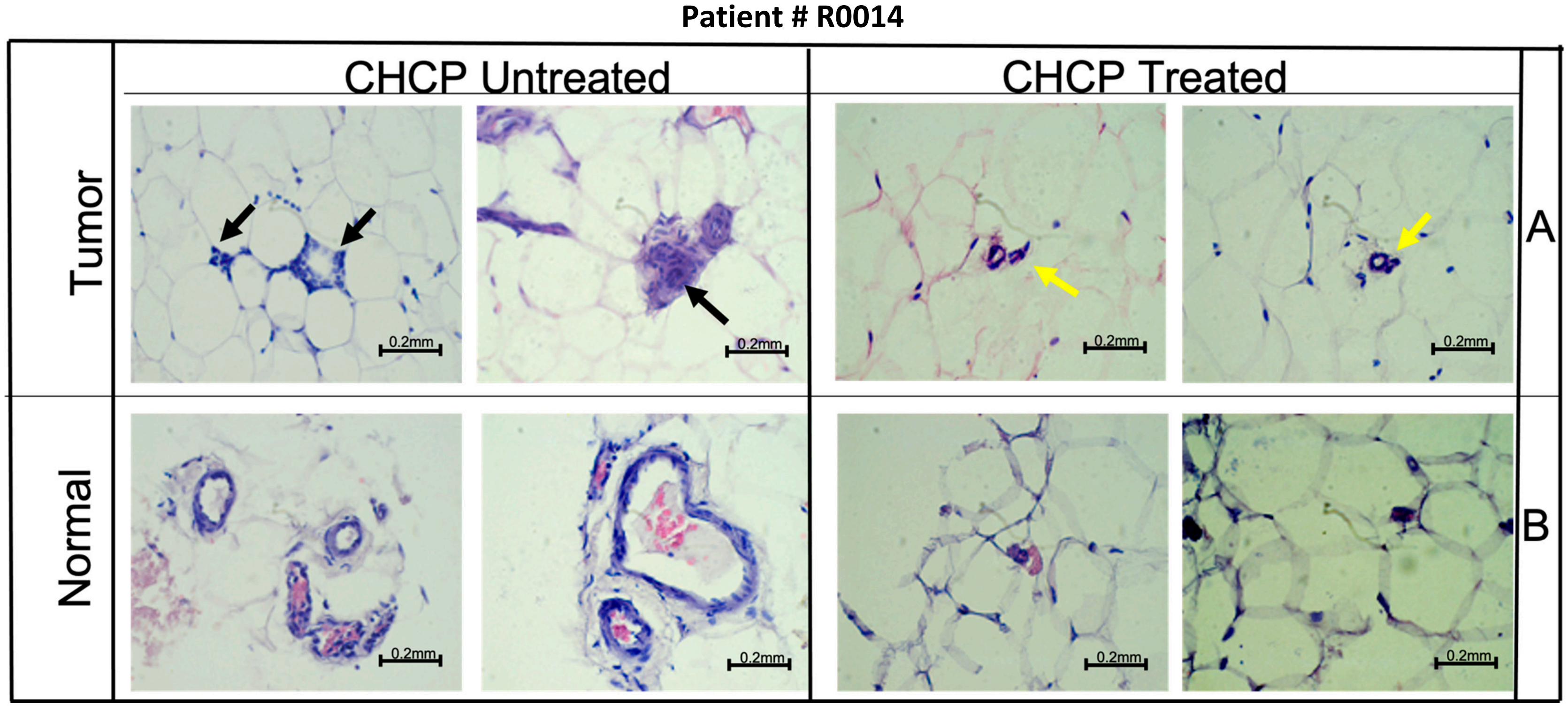

Figure S4. Histopathology of Patient Tissue Samples

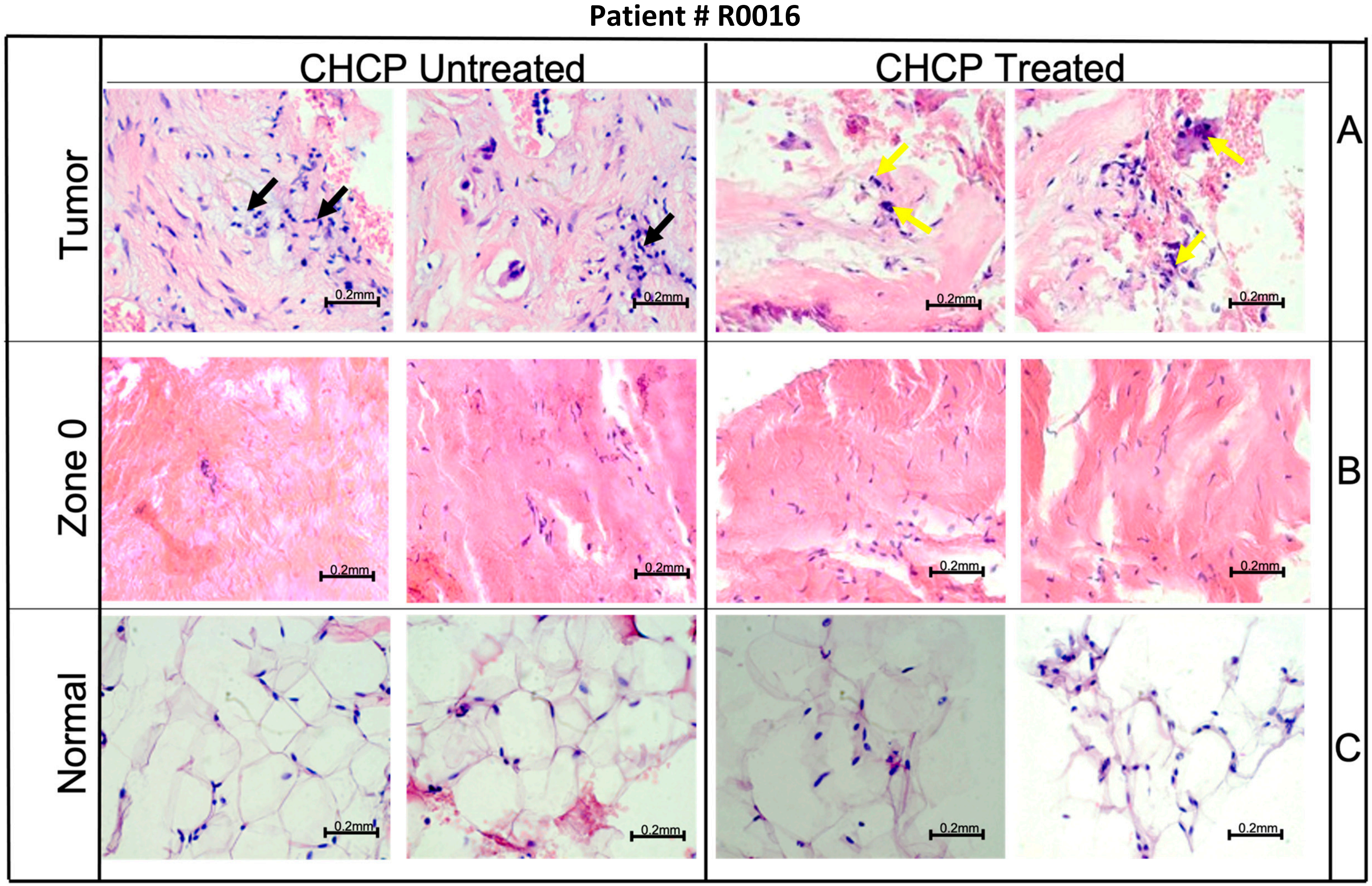

Figure S4. Histopathology of Patient Tissue Samples

Patient # R0017

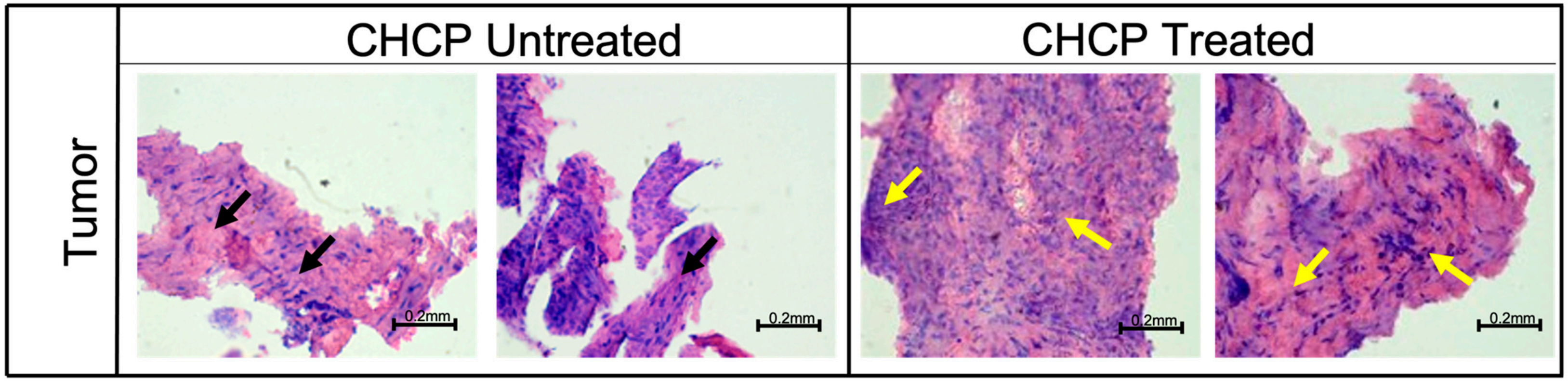

Figure S4. Histopathology of Patient Tissue Samples

Patient # R0018

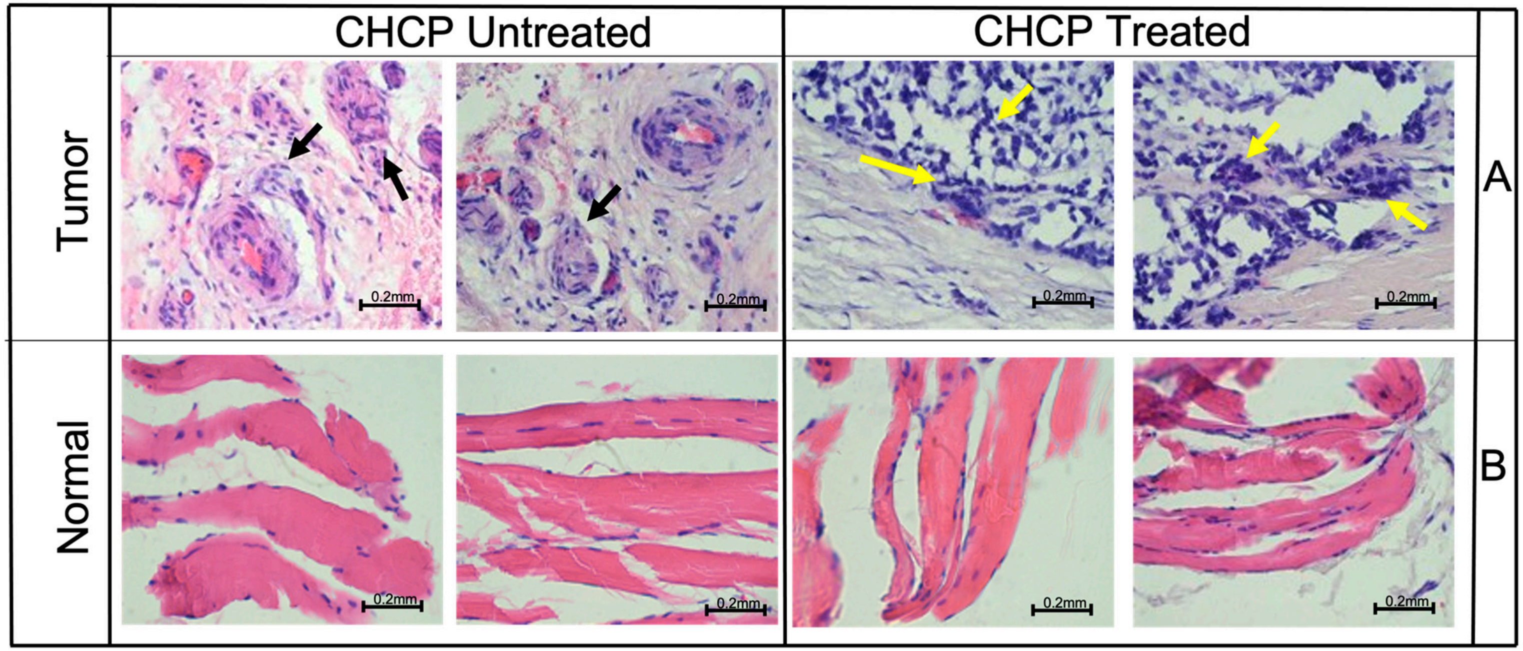

Figure S5. Primary cell culture of patient tumor samples. Tumor cells survival from the CHCP-treated tissues was significantly lower compared to the untreated tumor tissues in 8 out of 10 cases except for melanoma (R0010) and squamous cell carcinoma (R0013). Morphological changes to tumor cells after CHCP treatment were also observed in primary culture: fewer polygonal or rounded histiocyte-like shaped (R0003, R0009, R0014) or vacuolated physaliferous (R0011) cancer cells which occurs during cell death. Most cells survived from R0014 were tumor associated fibroblast cells. In two cases CHCP treatment did not significantly reduce the number of cancer cells or change the morphology (R0010 and R0013) because subjects R0010 and R0013 received sub-optimal CHCP treatment (6 min instead of 7 to 8 min) due to a malfunction of the connection between the cold plasma scalpel and CHCP generator.

Subject ID	Cancer type	Untreated tumor	CHCP-Treated tumor	Quantification of cells or colonies
R0002	Metastatic Recurrent Myxofibrosarcoma to the chest wall	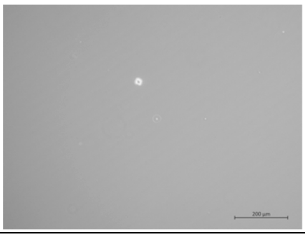	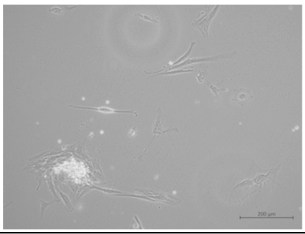	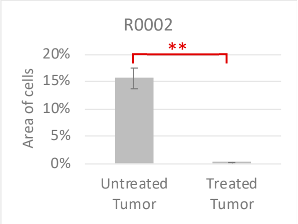
R0003	Metastatic Recurrent Breast Carcinoma to the right hip	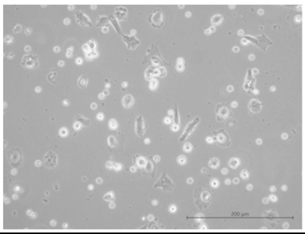	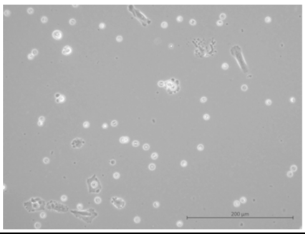	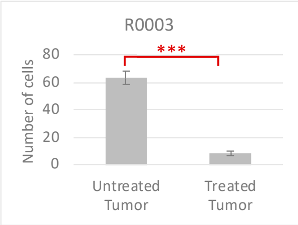
R0007	Metastatic Adenocarcinoma (Cholangiocarcinoma) to the left elbow/distal humerus	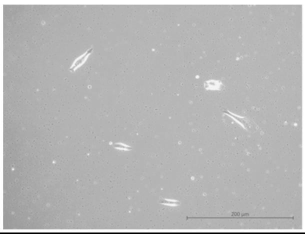	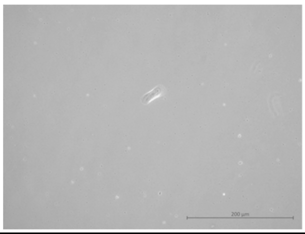	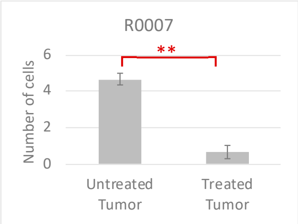
R0009	Metastatic Pleomorphic Sarcoma to the left distal femur	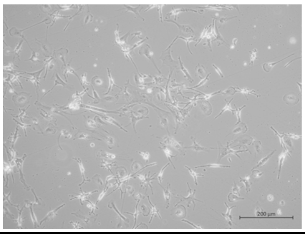	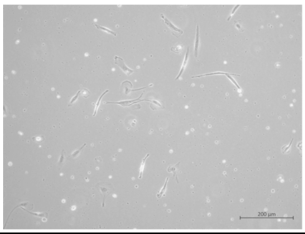	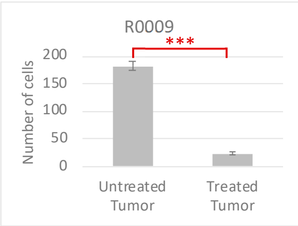

Figure S5. Primary cell culture of patient tumor samples.

<table>
<tr><th>Subject ID</th><th>Cancer type</th><th>Untreated tumor</th><th>CHCP-Treated tumor</th><th>Quantification of cells or colonies</th></tr>
<tr><td>R0011</td><td>Metastatic Recurrent Chordoma to the right gluteal (posterior thigh)</td><td>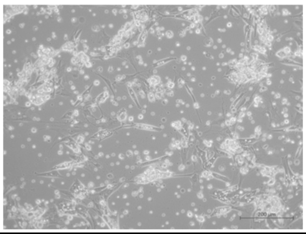</td><td>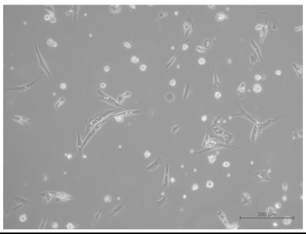</td><td>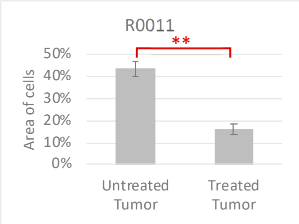
</td></tr>
<tr><td>R0010</td><td>Metastatic Recurrent Melanoma to the left pelvis</td><td>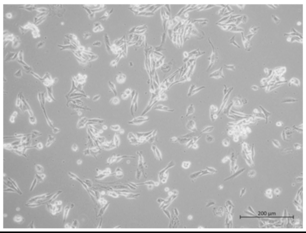</td><td>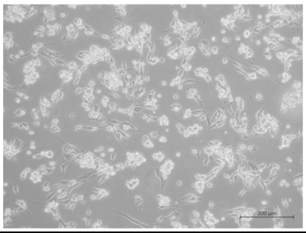</td><td>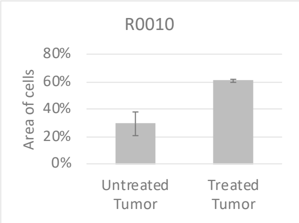
</td></tr>
<tr><td>R0013</td><td>Metastatic Recurrent Squamous Cell Carcinoma to the left hand and left axillary lymph node</td><td>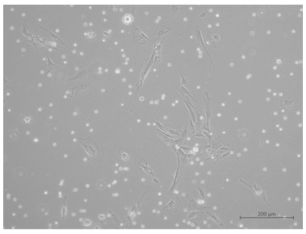</td><td>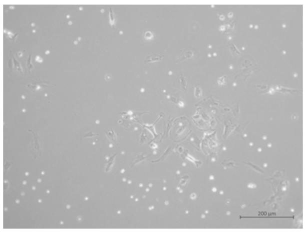</td><td>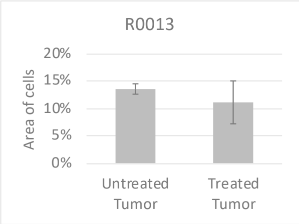
</td></tr>
<tr><td>R0014</td><td>Metastatic Angiosarcoma of the right Breast and the contralateral left breast axilla</td><td>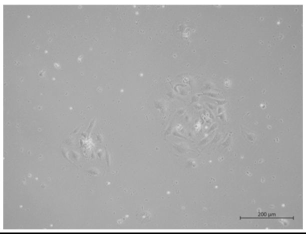</td><td>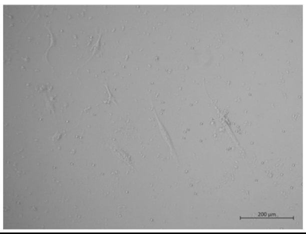</td><td>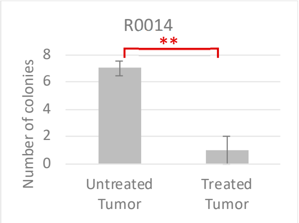
</td></tr>
<tr><td>R0017</td><td>Metastatic Desmoplastic Small Round Cell Sarcoma (DSRCS) of inguinal lymph node and testicle</td><td>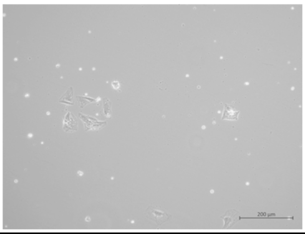</td><td>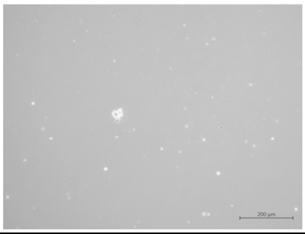</td><td>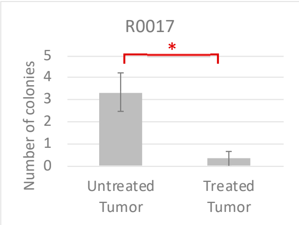
</td></tr>
<tr><td>R0018</td><td>Metastatic Recurrent Adenoid Cystic Carcinoma</td><td>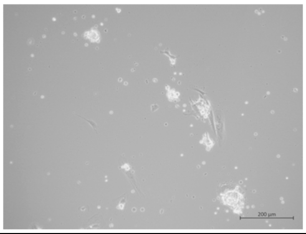</td><td>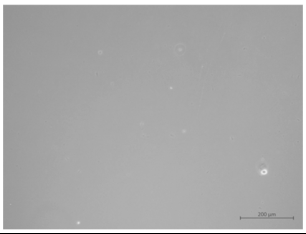</td><td>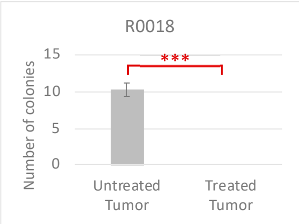
</td></tr>
</table>

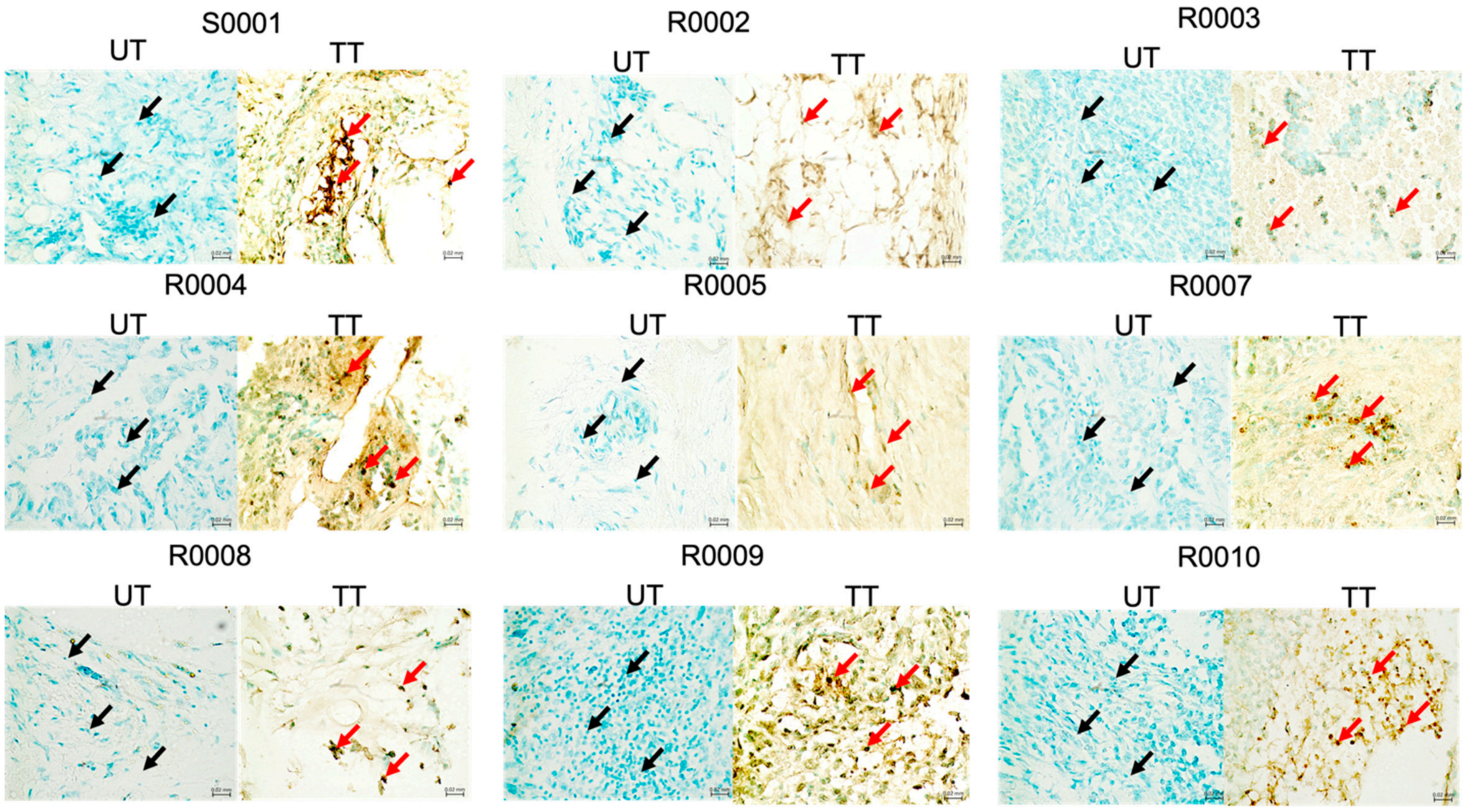

Figure S6. TUNEL Assay Analysis. Representative images of apoptosis analysis by TUNEL assay staining for patient tissue samples that were CHCP untreated or treated. Tissues are labeled: UT→ Untreated Tumor; TT→ Treated Tumor; Z0-UT→ Zone Zero Untreated; Z0-T→ Zone Zero Treated; Z1-UT→ Zone One Untreated; Z1-T→ Zone One Treated; N-UT→ Normal Untreated; N-T→ Normal Treated. Black arrows and red arrows point to untreated tumor cells and treated dead tumor cells respectively. Purple and Blue arrows point to untreated and treated normal cells respectively. Apoptotic cells were labeled with TdT and signals developed using DAB. No cell death visible with only Methylene Green counter stain. Most of CHCP untreated tissue sections do not show TdT signal. microscopic tumor cells were identified at the surgical margin site (Zone 0) in 5 patients (42%) (R0004, R0005,R0009 R0011 and R0012). Light Micrographs displaying the morphological Spectrum at 63X.

Figure S6. TUNEL Assay Analysis

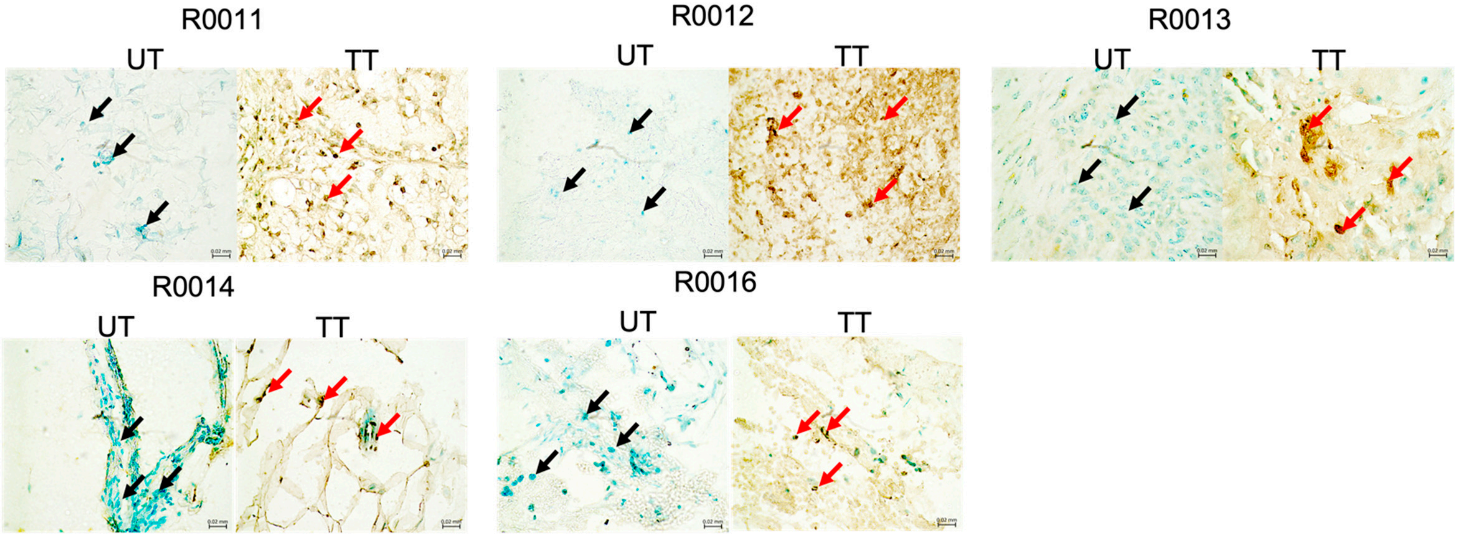

Figure S6. TUNEL Assay Analysis.

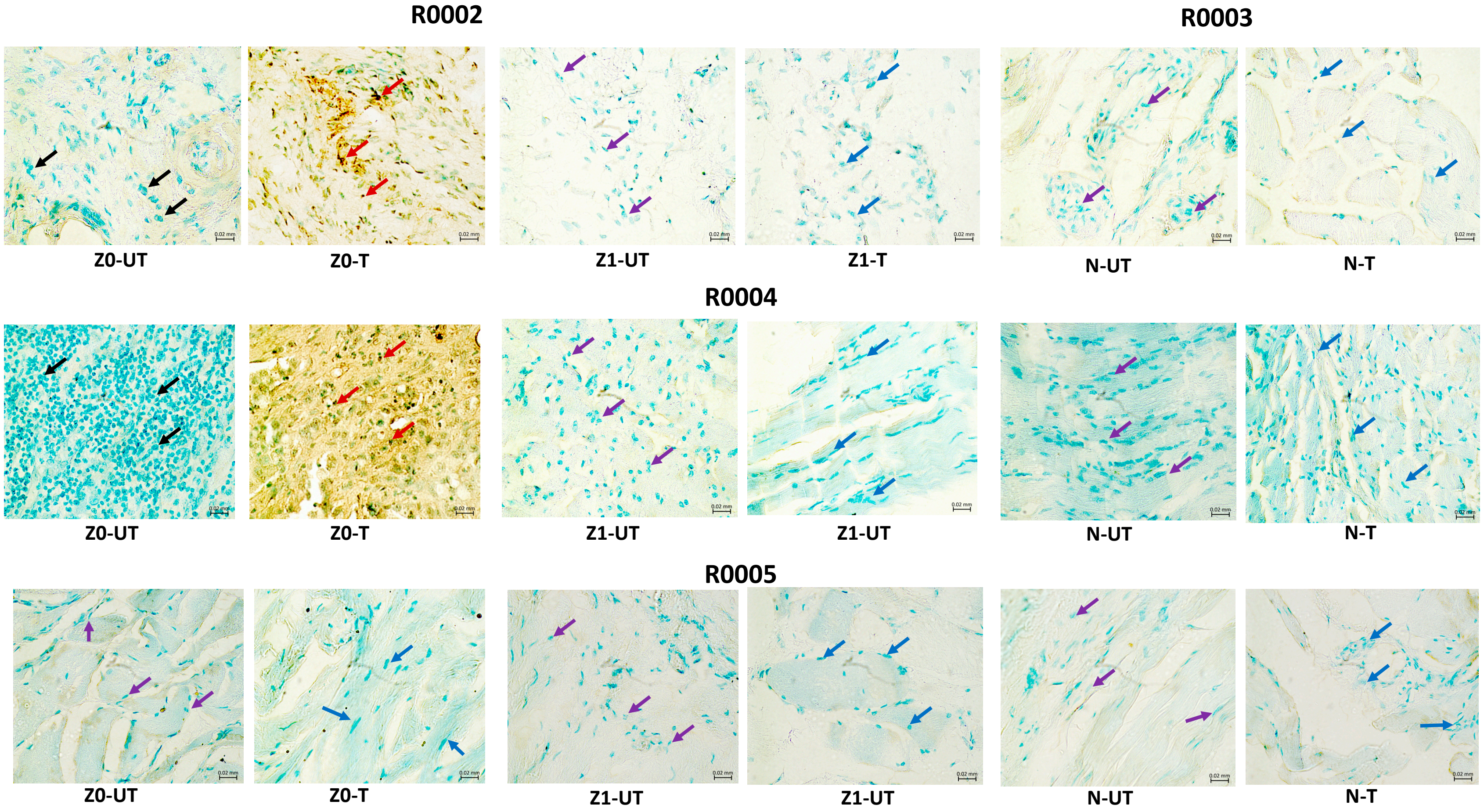

Figure S6. TUNEL Assay Analysis.

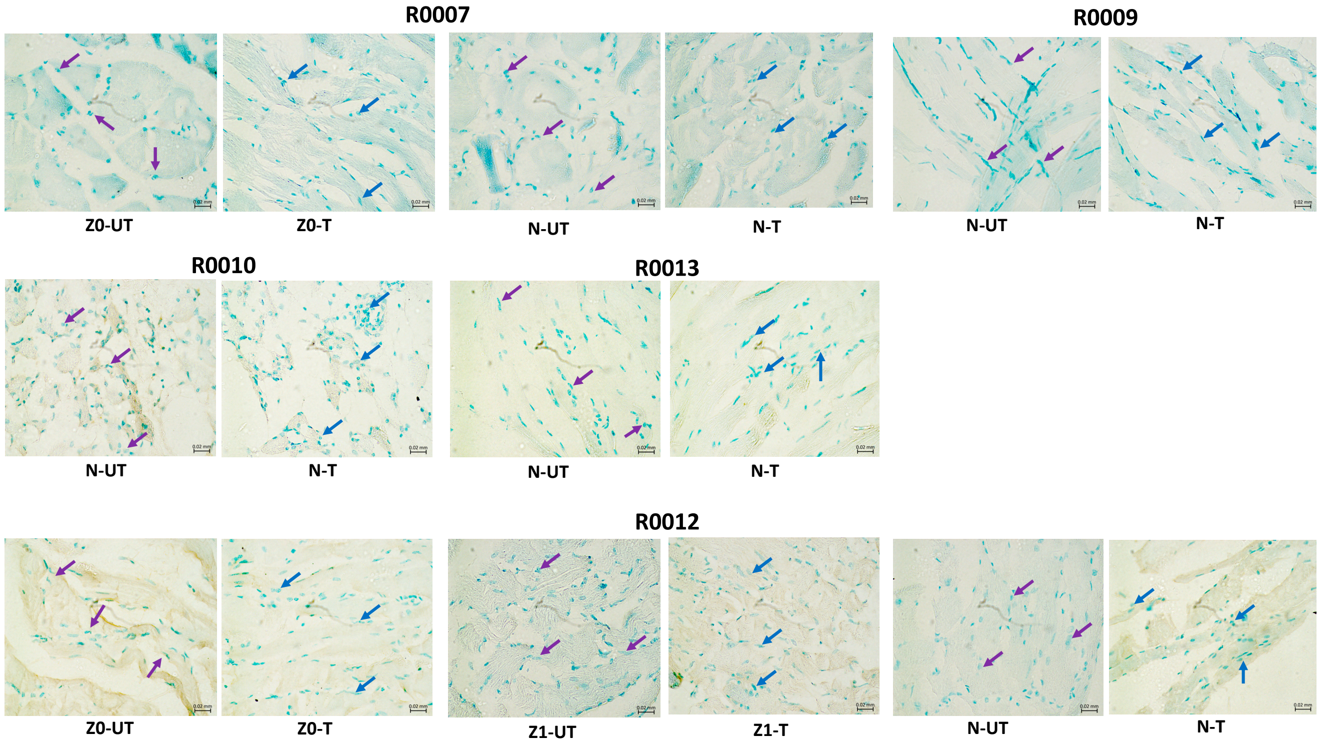

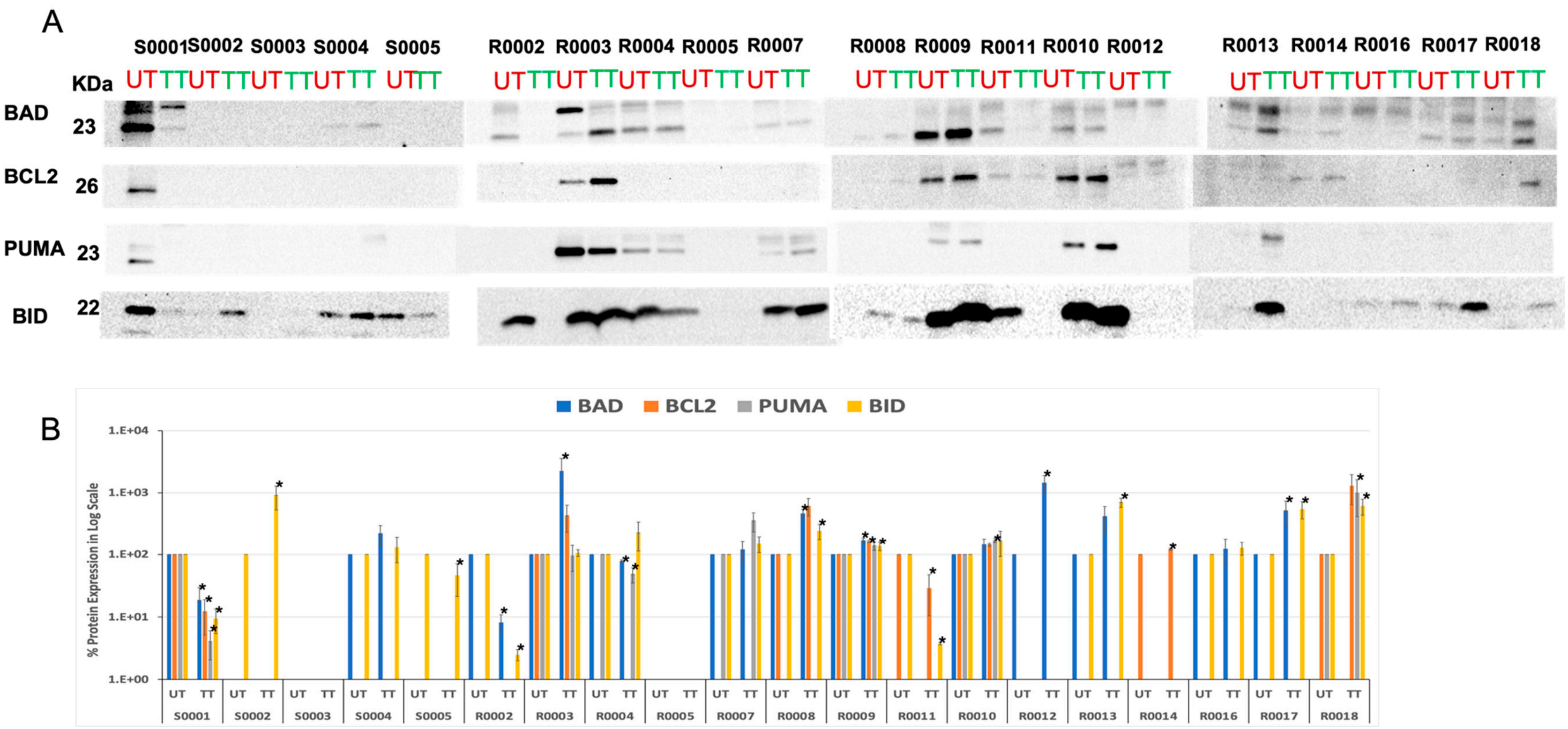

Figure S7. Western blot analysis for apoptotic markers after CHCP treatment on patient tumor samples. "A" showing the protein band of BAD, BCL2, PUMA and BID patient un-treated (UT) or treated (TT) tumor tissue samples. "B" showing the Bar graph for the quantification of protein bands. The data is represented by the SEM (n=3). (* $p < 0.05$, Student's t-test). PUMA was up regulated in Cholangiocarcinoma ($p< 0.051$) and Pleomorphic Sarcoma ($p < 0.069$). Apoptosis protein marker BAD was significantly up regulated in tumor CHCP treated samples: soft tissue sarcoma ($p < 0.00042$), breast carcinoma ($p < 0.028$), non-small cell lung adenocarcinoma ($p < 0.0015$), pleomorphic sarcoma ($p < 0.0056$), pleomorphic spindle cell sarcoma ($p < 0.019$) and desmoplastic small round cell sarcoma (DSRCS) ($p < 0.017$). PUMA was significantly up regulated in patient's treated tumor tissues in Melanoma ($p < 0.0028$) and DSRCS ($p < 0.028$). Anti-apoptotic marker BCL2 was down regulated in colon cancer ($p < 0.0061$) and up regulated in pleomorphic sarcoma ($p < 0.012$), recurrent melanoma ($p < 0.043$) and angiosarcoma ($p < 0.047$) patient's tumor CHCP treated samples. Original western blots attached in Excle.

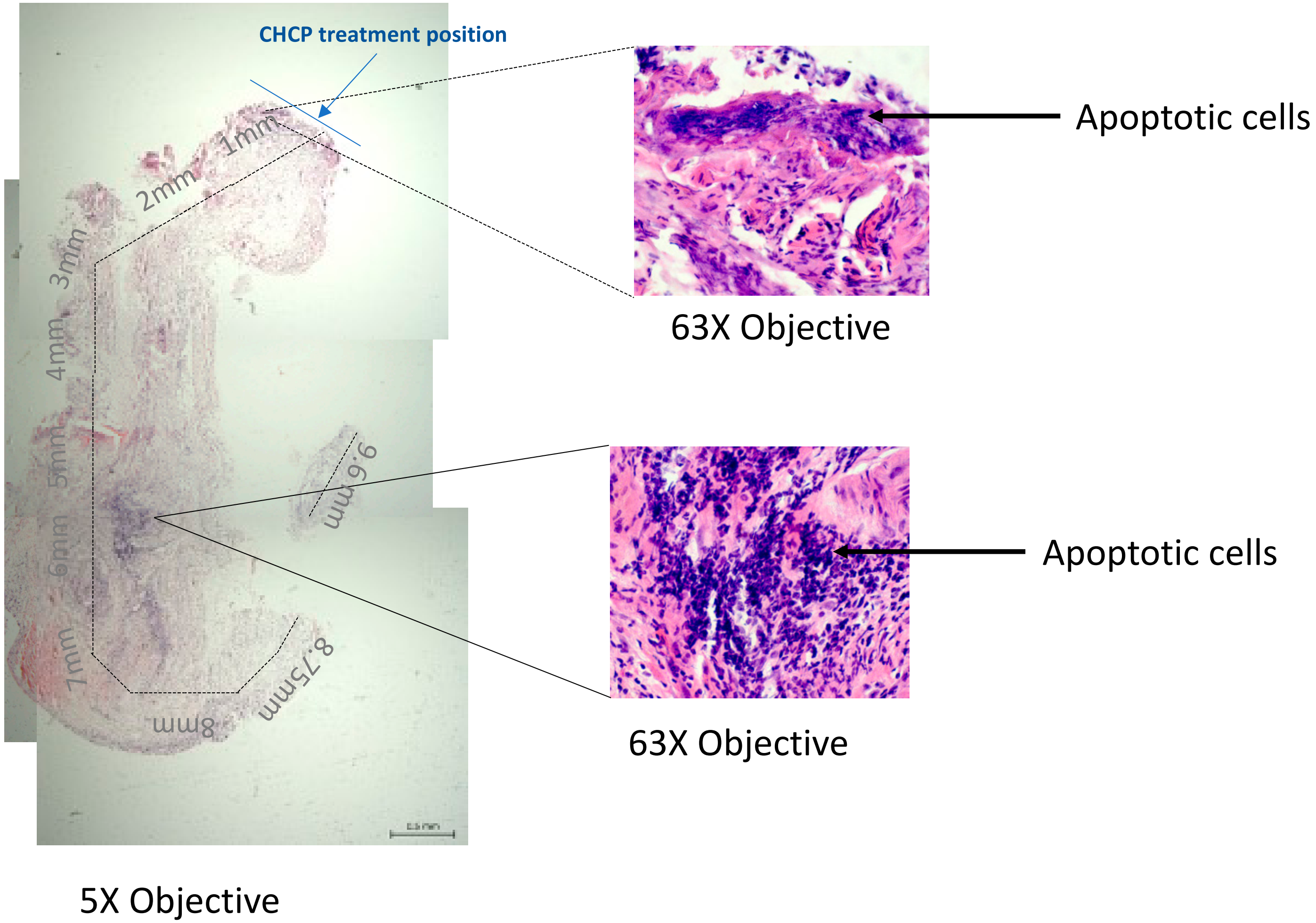

Figure S8. Tumor Cell Death at the surgical margin: Light micrographs displaying H & E staining of Patient R0004 (metastatic recurrent non-small cell lung adenocarcinoma to the left hip/upper end of femur) Zone 0, tissue (Surgical margins) samples with CHCP treatment.

Table S1. Gene profiling analysis. A summary of the results of a gene profiling study on seven clinical trial patients (IDE 190165). Untreated and *ex vivo* CHCP-treated tumor samples were collected and processed for RT-qPCR to analyze a panel of 111 genes. The table shows the number of genes which were upregulated (green arrow) or downregulated (red arrow) immediately after CHCP treatment, compared to untreated controls ($p < 0.05$).

Gene Profiling for IDE 190165

comparing untreated vs treated tumor tissue samples

				Tumor Type and Patient ID													
				Colon S0001		Ovarian S0002		Anal S0003		Mesothelioma S0004		Lung R0004		Sarcoma R0009		Melanoma R0010	
**Category of Genes**			# of genes screened	# of genes significantly upregulated	# of genes signficantly down-regulated	# of genes significantly upregulated	# of genes signficantly down-regulated	# of genes significantly upregulated	# of genes signficantly down-regulated	# of genes significantly upregulated	# of genes signficantly down-regulated	# of genes significantly upregulated	# of genes signficantly down-regulated	# of genes significantly upregulated	# of genes signficantly down-regulated	# of genes significantly upregulated	# of genes signficantly down-regulated
**Apoptosis**	Induction of Apoptosis	Death Receptors	6	↑ 4	0	0	↓ 1	0	↓ 1	↑ 1	↓ 3	↑ 2	0	0	↓ 1	↑ 1	↓ 2
		DNA Damage & Repair	4	↑ 3	0	0	0	0	0	↑ 2	↓ 1	↑ 1	0	0	↓ 2	0	↓ 1
		Extracellular Signals	1	↑ 1	0	0	0	0	↓ 1	↑ 1	0	↑ 1	0	0	0	0	↓ 1
	Caspases & Caspase Regulation	Initiator Caspases	4	↑ 4	0	0	↓ 1	↑ 2	0	↑ 1	↓ 1	0	0	0	↓ 3	0	↓ 2
		Effector Caspases	3	↑ 3	0	0	0	↑ 1	0	0	0	0	0	0	↓ 1	0	↓ 1
		Inflammatory Caspases	4	↑ 2	0	0	↓ 1	↑ 1	0	↑ 1	↓ 1	↑ 1	0	0	↓ 2	0	↓ 1
		Regulation	1	0	↓ 1	0	0	↑ 1	0	0	0	0	0	0	↓ 1	0	0
		Activation	4	↑ 2	0	0	0	0	0	0	0	↑ 1	0	0	↓ 3	0	0
		Inhibition	2	↑ 1	0	0	0	0	↓ 1	↑ 1	↓ 1	↑ 1	0	0	↓ 2	0	0
	Regulation of Apoptosis	Positive Regulation	9	↑ 9	0	0	↓ 1	↑ 1	0	↑ 3	↓ 1	↑ 3	↓ 1	↑ 1	↓ 5	↑ 1	↓ 1
		Negative Regulation	20	↑ 18	0	0	↓ 4	↑ 4	↓ 6	↑ 7	↓ 6	↑ 8	0	↑ 1	↓ 11	↑ 3	↓ 7
**Oxidative Stress**	Reactive Oxygen Species (ROS) Metabolism	Superoxide Dismutases (SOD)	2	↑ 1	↓ 1	0	0	0	↓ 2	0	↓ 1	0	0	0	↓ 2	↑ 1	0
		Superoxide Metabolism	7	↑ 3	0	↑ 1	0	↑ 1	↓ 2	0	↓ 4	↑ 3	0	0	↓ 4	0	↓ 2
		ROS Metabolism	1	↑ 1	0	0	↓ 1	0	0	0	0	0	0	0	↓ 1	0	0
		Oxidative Stress Response	26	↑ 22	0	0	↓ 5	↑ 1	↓ 5	↑ 7	↓ 5	↑ 11	↓ 2	↑ 2	↓ 9	↑ 2	↓ 4
**Other Genes**	Immune Checkpoint		10	↑ 9	0	0	↓ 5	0	↓ 3	↑ 4	↓ 4	↑ 6	0	0	↓ 9	0	↓ 2
	T-Cell Regulation		4	↑ 2	0	0	↓ 2	↑ 2	↓ 2	0	↓ 1	↑ 1	0	0	↓ 7	0	0
	Other Tumor Promoter Genes		3	↑ 3	0	0	0	↑ 1	0	0	↓ 1	↑ 2	0	↑ 1	↓ 2	↑ 1	↓ 2
**Total # of Genes Differentially Expressed (p < 0.05)**				↑ 88	↓ 2	↑ 1	↓ 21	↑ 15	↓ 23	↑ 28	↓ 30	↑ 41	↓ 3	↑ 5	↓ 65	↑ 9	↓ 26
**Total # of Genes Screened**			111														
